# Role and Mechanism of Galactose-Alpha-1,3-Galactose in the Elicitation of Delayed Anaphylactic Reactions to Red Meat

**DOI:** 10.1007/s11882-019-0835-9

**Published:** 2019-01-23

**Authors:** Christiane Hilger, Jörg Fischer, Florian Wölbing, Tilo Biedermann

**Affiliations:** 1 Department of Infection and Immunity, Luxembourg Institute of Health (LIH), 29, rue Henri Koch, L-4354 Esch-sur-Alzette, Luxembourg; 2 Department of Dermatology, Eberhard Karls University Tübingen, Tübingen, Germany; 3 Department of Dermatology and Allergy Biederstein, Technical University of Munich, Munich, Germany; 4 Clinical Unit Allergology, Helmholtz Zentrum München, German Research Center for Environmental Health GmbH, Neuherberg, Germany

**Keywords:** Alpha-gal, Carbohydrate, Food allergy, Red meat allergy, Tick

## Abstract

**Purpose of Review:**

The alpha-Gal (α-Gal) syndrome is characterized by the presence of IgE antibodies directed at the carbohydrate galactose-alpha-1,3-galactose (α-Gal). In this article, we review the presence of α-Gal in food and non-food sources; we discuss the evolutionary context of the antibody response to α-Gal and highlight immune responses to α-Gal and other carbohydrates.

**Recent findings:**

IgE antibodies have been associated with delayed allergy to red meat. In addition to food, drugs, and other products of animal origin are increasingly perceived as a risk for patients sensitized to α-Gal. The link between tick bites and anti-α-Gal IgE-antibody production that has been established first by epidemiological studies has now been confirmed in mouse models.

**Summary:**

The anti-α-Gal immune response is complex and characterized by a unique feature. IgM and IgG antibodies have been found to confer protection against pathogens whereas the IgE-response to α-Gal is detrimental and causes severe reactions upon exposure to mammalian meat and other products.

## Introduction

The starting point for the discovery of the α-Gal syndrome was the approval of cetuximab, a chimeric mouse-human IgG1 monoclonal antibody directed against the epidermal growth factor receptor, for the treatment of colorectal and squamous-cell cancer by the US Food and Drug Administration (FDA) in 2003. In contrast to previous experiences in the registration trials, a high rate of hypersensitivity reactions occurred upon the first application of cetuximab in the southeastern US federal states [1]. In the course of further examinations, Chung et al. identified specific IgE antibodies that surprisingly did not recognize a particular peptide sequence but a carbohydrate structure, galactose-alpha-1,3- galactose (α-Gal) [2]. α-Gal is a common component in glycan structures of mammals. But there is an important exception due to a unique evolutionary event about 20–28 million years ago. In ancestral Old World monkeys and apes, the gene for the enzyme α-1,3-galactosyltransferase that is essential for the synthesis of α-Gal was inactivated [3]. Therefore, humans and recent Old World primates do not express α-Gal and this structure is highly immunogenic for them. In the late 80s, transplant immunology had already identified natural anti-α-Gal antibodies, as IgG, IgM, and IgA isotypes, as the most abundant antibodies in humans. For xenotransplantation, i.e., transplantation of organs from pigs into humans, these antibodies represent a major immune barrier [3]. A new aspect of the α-Gal immune response was the circumstance that an IgE isotype of anti-α-Gal antibodies with a high anaphylactic potential had been found [4].

## α-Gal and Red Meat Allergy

**Fig. 1:**
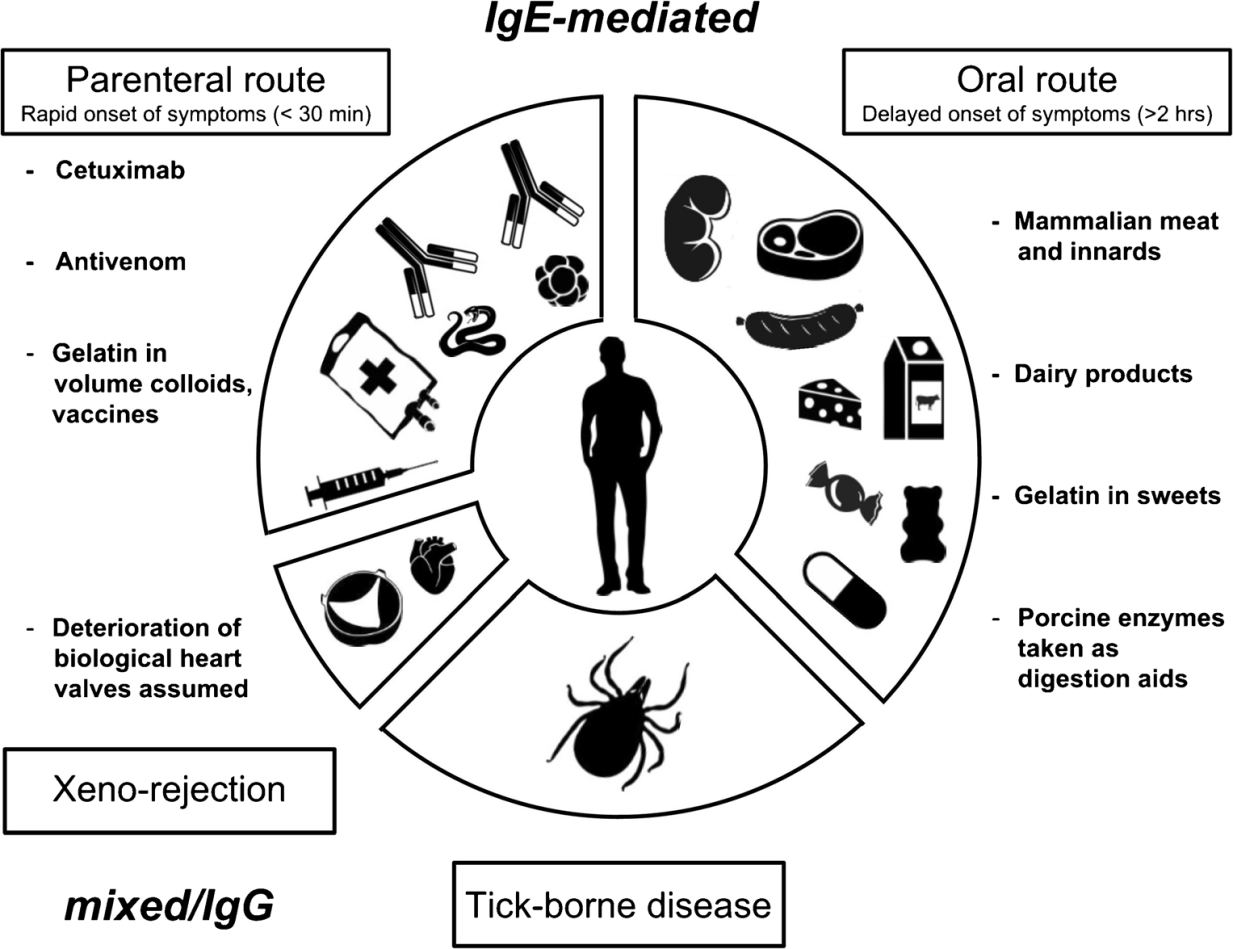
α-Gal syndrome. The α-Gal syndrome describes an IgE-mediated allergy to the disaccharide galactose-α-1,3-galactose (α-Gal). Base arc—tick bites are assumed to be the most frequent and most important primary sensitization source to α-Gal, and α-Gal syndrome is the first allergy classified as tick-borne disease. Left upper arc—administration of drugs derived from mammalian cells and tissue (like cetuximab, antivenom, gelatin in colloids or vaccines) can induce drug allergy. Left lower arc—as part of xeno-rejection, deterioration of biological heart valves is assumed. Right arc—consumption of mammalian muscle meat and innards, but also dairy products, gelatin in sweets or porcine pancreatic enzymes in digestion aids can induce food allergy

Upon examination of patients with anaphylaxis to cetuximab, Collins et al. discovered a new form of allergy to red meat in 2009 [5]. In contrast to common experience in food allergy, these allergic reactions have as unique feature a delayed onset of effects of 3–6 h after consumption of mammalian meat (Fig. 1). The clinical severity of these type-I reactions can vary and ranges from urticaria/angioedema to life-threatening anaphylaxis with hypotension, shock, and unconsciousness. The reason for this delay of onset is not yet understood, but an association with digestion processes, especially fat digestion, is assumed [6]. Clinical findings in oral challenges show [7•] that according to the concept of food-dependent exercise-induced anaphylaxis (FDEIA) [8], endogenous and exogenous factors (e.g. alcohol, physical exercise, non-steroid analgesic, infections, and menstruation) are able to enhance the allergic reaction to mammalian meat. This observation can explain why quite a lot of α-Gal IgE-positive individuals have no evidence of allergic reactions to mammalian meat as their exposure is probably below the threshold for induction of symptoms in the absence of cofactors [9]. But these individuals are under potential risk to develop anaphylaxis upon exposure to a certain amount of α-Gal in combination with relevant augmenting factors [9]. In foods, α-Gal is not only found in meat products. Dairy products, like milk, cream, or cheese also contain small amounts of α-Gal (Fig. 1). Gelatin is another source for α-Gal and widely used in foods as gelling and thickening agent, for example in sweets, cake glace, or fat-reduced dairy products (Fig. 1) [10, 11••]. Accordingly, clinical observations indicate that these α-Gal sources are tolerated in some α-Gal allergic patients, but not in highly allergic individuals [5, 10, 12]. The consumption of mammalian innards, like pork kidney, takes on a special position in α-Gal syndrome as symptoms often are more severe and occur more rapidly (Fig. 1). Upon consumption of pork kidney, an immediate onset of anaphylaxis instead of the typical delayed onset is regularly observed [13]. Therefore, innards are regarded as the most potential α-Gal source. It is commonly assumed that this is linked to a higher content in α-Gal in innards compared to muscle meat [7•, 13]. In Europe, α-Gal syndrome patients are described, who only react after consumption of mammalian innards, but not after consumption of mammalian muscular meat [12]. Angiotensin I-converting enzyme (ACE I) and aminopeptidase N (AP-N), both highly glycosylated proteins of urogenital tissues, were identified as major IgE-binding molecules in pork kidney [14•]. The isolated proteins were able to induce basophil activation in patients with α-Gal syndrome, and IgE-binding was shown to be directed to α-Gal epitopes carried by both proteins. Two other studies identified several IgE-binding proteins carrying α-Gal epitopes in beef meat by two-dimensional gel electrophoresis and peptide mass fingerprinting techniques [15, 16].

## The Association with Ticks

The geographic outlined accumulation of hypersensitivity in the southeastern US federal states, and the fact that patients developed anaphylaxis on first contact with cetuximab indicated that the patients were presensitized and inducing environmental factors were searched for [17]. A report from Australia, describing a correlation between allergic local reactions to tick bites and allergy to red meat, brought ticks in the focus of US researchers [17, 18]. The epidemiological matching of occurrence of anaphylaxis to cetuximab with an endemic area of the rocky-mountain spotted fever, a tick-borne disease mainly transmitted by *Amblyomma americanum*, supported the tick-hypothesis. Today, we know that in addition to *Amblyomma spp.*, various other tick genera, like *Haemaphysalis spp.* and *Ixodes spp.*, are involved as well [19, 20] (Fig. 1). The presence of α-Gal in ticks could be demonstrated by ELISA, immunoblotting techniques, and immunohistochemical staining in various tick species [19, 20]. Recently, Araujo et al. showed that α-1,3-galactosyltransferase-deficient mice could be sensitized to α-Gal by tick saliva of *Amblyomma sculptum* [21••]. Therefore, it is commonly assumed that tick bites are the most frequent and most important primary sensitization source to α-Gal and α-Gal syndrome is the first allergy classified as tick-borne disease. Recently, chiggers, mites of the *Trombiculidae* family at their larval stage, have been reported to be associated with sensitization to α-Gal and delayed allergy to red meat in three patients [22]. Ticks do not only serve as a vector for sensitization, but tick bites can also be associated in some cases with anaphylaxis. Tick-induced anaphylaxis is most often observed in Australia during removal of ticks [23]. Local allergic reactions around the bites, presenting as erythematous papules or plaques itching for more than 10 days, are regularly observed in α-Gal-sensitized individuals after tick bites [17]. While initially the α-Gal syndrome was seen as local allergy in the southeastern US federal states, we know now that the α-Gal syndrome occurs on all continents and has a global health impact.

As anti-α-Gal IgE is an environmental type-I sensitization, its prevalence in a human population depends on the degree of exposure to ticks. In an epidemiological survey among hunters and forest workers, a high prevalence of type-I sensitizations to α-Gal of 35.0% was observed (α-Gal-sIgE levels > 0.10 kUA/L) [24•]. A history of α-Gal syndrome was found in 8.6% of the α-Gal-sIgE-positive participants with levels > 0.35 kUA/L [24•]. In rural European areas, the prevalence of type-I sensitizations to α-Gal can be up to 24.7% (α-Gal-sIgE levels > 0.10 kUA/L) [25]. In contrast, in general adult populations living in an urban environment, the prevalence ranges between 5.5 and 8.1% (α-Gal-sIgE levels > 0.10 kUA/L) [26]. This high prevalence of α-Gal-sIgE levels challenges the diagnosis of the α-Gal syndrome. Skin prick tests using meat extracts often give false negative results whereas prick-to-prick tests with pork or beef kidney or intradermal testing with gelatin colloid are sensitive, but elaborate in handling [5, 7•]. α-Gal-sIgE levels are not predictive for the clinical relevance regarding the consumption of mammalian meat or α-Gal containing drugs [5, 7•, 11••]. Therefore, diagnosis of the α- Gal syndrome strongly relies on clinical history and food challenges are warranted to determine the clinical relevance [11, 27•]. A recent study has shown that the basophil activation test is of added value for differentiation between patients with α-Gal syndrome and asymptomatic α-Gal sensitization [28•].

The closest human carbohydrate to α-Gal is blood antigen B, a fucosylated Galα1 → 3Gal epitope. The proposed hypothesis that individuals with blood type B have reduced susceptibility to type I sensitization to α-Gal and reduced α-Gal-sIgE levels is controversial. The assumption that individuals with blood type B have a lower susceptibility to develop type-I-sensitizations to α-Gal is mainly supported by the case series of Hamsten et al. where the prevalence of B blood types was 3.6-fold lower than expected in comparison to the Swedish general population [29]. In an epidemiological survey in a highly tick-exposed population, this finding could not be reproduced [30]. In addition, Galili et al. could not find quantitative differences of natural anti-α-Gal IgG antibodies in different human blood groups [31].

## Evolutionary Aspects of the Human Anti-α-Gal Response

**Fig. 2:**
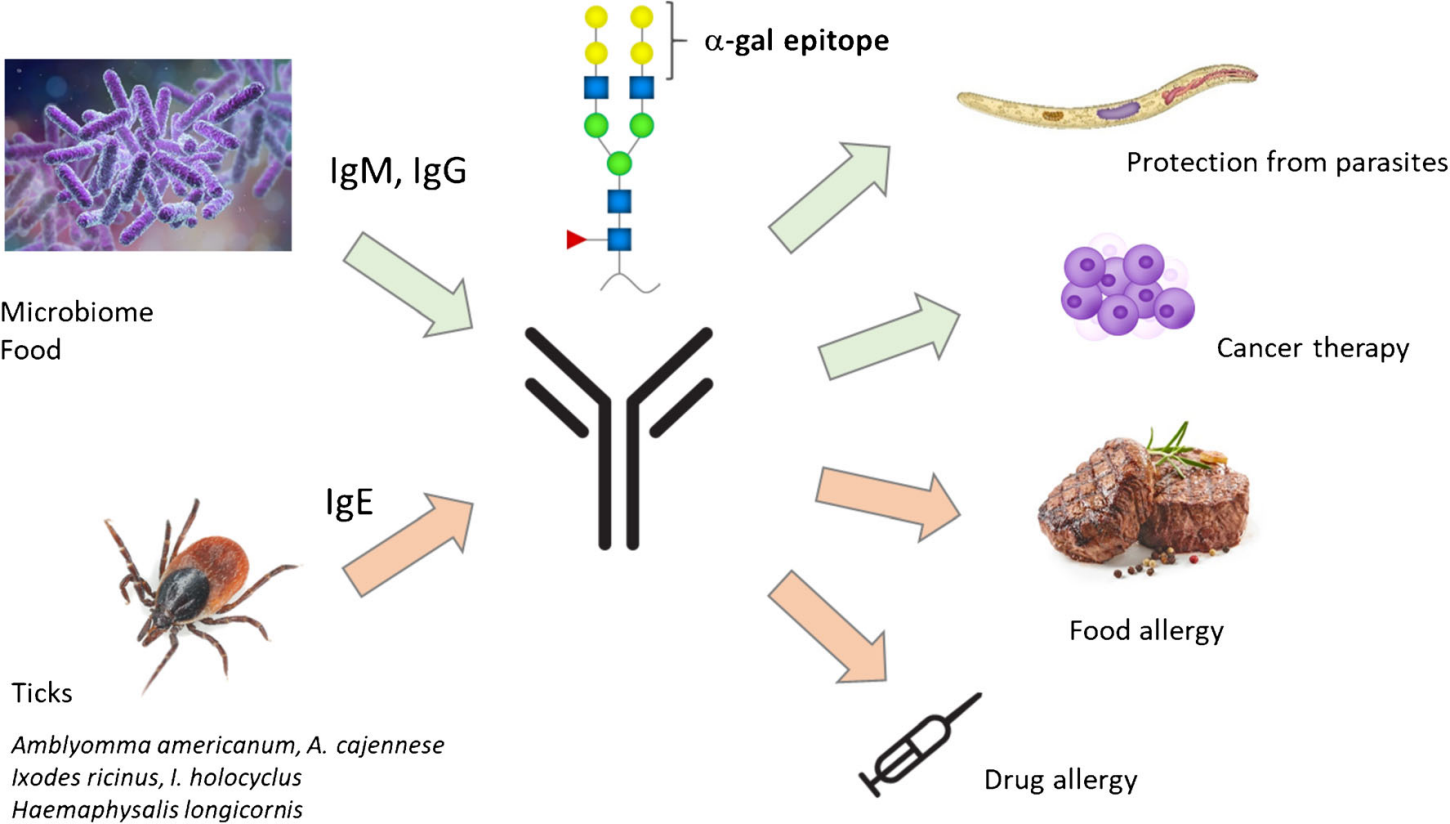
Anti-α-Gal antibodies: friend or foe? It is commonly assumed that IgM and IgG antibodies are generated by continuous stimulation by the intestinal microbiome and probably also by food. Bites of different hard ticks have been associated with the production of IgE antibodies directed to α-Gal. Whereas IgE antibodies are detrimental and responsible for anaphylactic reactions to food and drugs, IgG and IgM antibodies seem to play a role in protection from parasites and possibly other pathogens. The insertion of α-Gal glycolipids into tumor cells has been investigated in pre-clinical models of cancer immunotherapy. A typical α-Gal carbohydrate structure as e.g. present on cetuximab [36] is shown above the antibody. Yellow circle: galactose; green circle: mannose; blue square: *N*-acetylglucosamine; red triangle: fucose

The α-1,3-galactosyltransferase (α1,3GT) gene named GGTA1 is present in all mammals, but in humans, apes, and Old World Monkeys, it is inactivated by frame-shift mutations that truncate the expressed protein and abolish its catalytic activity [32]. α1,3GT catalyzes the transfer of a galactose residue with an α-1,3 linkage, on terminal lactosaminide (Gal-β-1,4-GlcNAc-R) disaccharide on glycoproteins and glycolipids, generating terminal α-Gal. The gene GGTA1 seems to have emerged early during mammalian evolution since it is also active in marsupials. It is suggested that the inactivation of the gene was the result of a strong evolutionary pressure. Primates with an inactive gene would not synthesize α-Gal epitopes but would be able to develop anti-α-Gal antibodies which would have been of advantage in fighting pathogens expressing α-Gal. It has indeed been shown that enveloped viruses produced in animal cells or human cells engineered to contain active α1,3GT contain multiple α-Gal epitopes on their surface, rendering them more sensitive to lysis by human anti-α-Gal antibodies and complement [33, 34]. Earlier observations have reported that patients with leishmaniasis and patients with Chagas’ disease had markedly elevated titers of anti-α-Gal antibodies, raising the possibility of immunogenic stimulation by the respective parasites [35]. The presence of α-Gal epitopes on *Trypanosoma cruzi*, *Leishmania braziliensis* and *L. mexicana* parasites was confirmed by antibody and lectin staining, inhibition, and deglycosylation experiments [35]. Thus, the human anti-α-Gal antibody response may contribute to the natural defense against various parasites (Fig. 2).

A recent study addressed this question by using an α1,3 GT knockout (Ggta1 KO) mouse model [37••]. Mice were inoculated with the human pathobiont *E. coli* O86:B7 which has been shown to induce anti-α-Gal IgG and IgM antibodies in Ggta1 KO mice [38]. Circulating anti-α-Gal antibodies were able to target *Plasmodium sporozoites* immediately after inoculation in the skin and conferred protection from malaria transmission. In line with these results, the authors showed that in malaria-endemic areas, high levels of anti-α-Gal IgM antibodies are associated with a decreased risk of malaria transmission [37••]. Similar results were obtained in a study by Cabezas-Cruz and colleagues [39]. The levels of anti-α-Gal IgM and IgG antibodies were higher in healthy individuals than in individuals infected with *Plasmodium falciparum* or *Mycobacterium tuberculosis* [39]. These findings raise the question if vaccination approaches including the α-Gal epitope could be successful in conferring protection to malaria and possibly to other vector-borne diseases [40]. Two α-Gal vaccines against *Leishmania* infection have been tested in the Ggta1 KO mouse model. Both approaches, a neoglycoprotein carrying synthetic α-Gal epitopes and a virus-like particle carrying multiple α-Gal epitopes, were able to significantly reduce parasite burden in infected mice [41, 42].

Anti-α-Gal IgG antibodies are highly abundant in human serum, estimated at 30 to 100 μg/ml [43]. They cross the placenta and are detected in high titers in cord blood. Antibody levels are at lowest between 3 and 6 months, then increase gradually and reach titers comparable to adults between 2 and 4 years [44]. This high level of anti-α-Gal antibodies at early age raises the question about the source of the antigenic stimulus. Earlier studies have shown that human anti-α-Gal antibodies interact with a variety of *Escherichia coli*, *Klebsiella*, and *Salmonella* strains [45]. The fact that some of the *E. coli* and *Klebsiella* strains were obtained from normal human stool samples supports the hypothesis that bacteria of the intestinal microbiome would provide the antigenic stimulus for a continuing production of anti-α-Gal antibodies (Fig. 2). The spectrum of anti-Gal specificity however is dependent on the individual’s blood type. The α-Gal epitope is very similar to the B blood group antigen where a fucose is attached to the second galactose (Gal-α1,3 (Fuc-α1,2)-Gal). Anti-α-Gal antibodies purified from human O or A serum also recognized the B-antigen. On the contrary, IgG isolated from donors with AB or B blood group are not able to recognize the B-antigen because of immune tolerance and they have a more specific α-Gal response [46].

## α-Gal Is Present in Many Non-Food Sources

The list of drugs obtained from mammalian cells or tissues with specific risks for α-Gal sensitized individuals has increased over the years. Genetically engineered therapeutic antibodies play an important role in medicine, namely oncology or rheumatology. Luckily, with the exception of cetuximab, till now, no other therapeutic antibody conferring a comparable risk to α-gal sensitized individuals was found (Fig. 1) [47].

The α-Gal epitope of cetuximab is located in the Fab region which is of mouse origin [47]. α-Gal has also been identified in the Fc region of cetuximab and other therapeutic antibodies produced in mammalian cells such as SP2/O and NS0, but these were not recognized by IgE antibodies of α-Gal sensitized individuals [47]. On mAbs produced in CHO cells, α-Gal was undetectable. The risk of an anaphylactic reaction to those antibodies and to antibodies carrying the α-Gal epitope on the Fc part of the antibody was estimated to be very low. However, a recent case reported an anaphylactic reaction to infliximab, a monoclonal antibody carrying an α-Gal epitope on the Fc part and produced in SP2/0 cells [48].

The α-Gal epitope is also present on other pharmaceuticals and products of animal origin such as gelatin, antivenoms, and bioprosthetic heart valves. Gelatin is obtained by hydrolysis of collagen, a product obtained from bones and connective tissue of animals. The product is not only used in processed food and sweets such as desserts and gummy bears, but it is also used in vaccines and in gelatin colloids as plasma expander. The α-Gal epitope was detected on the collagen a-1 (VI) chain and in gelatin colloids [11••, 16]. Clinical reports include allergic reactions to gummy bears, a vaginal gelatin capsule, and vaccines with high gelatin content such as zoster vaccine which contains 15.58 mg per 0.65 ml dose [10, 49–52]. However, not all patients with α-Gal syndrome react to zoster vaccine [52, 53]. Allergic symptoms upon intravenous administration of gelatin colloids, intramuscular injection of vaccines, or intravaginal application of gelatin capsules appear within the first 30 min whereas upon oral administration of gelatin, symptoms appear delayed [10, 11••, 49–51].

Antivenoms are polyclonal antibody preparations obtained by immunization of horse or sheep with snake venom. In order to reduce the total load of administered protein, some antivenom formulations are enzymatically digested to produce divalent or monovalent immunoglobulin fragments (F (ab’)2/Fab). The Fab fragments of such preparations have been shown to carry the α-Gal epitope [54], and a case of hypersensitivity has been reported recently in an α-Gal-sensitized patient [55]. Recently, porcine enzyme preparations taken orally for digestion aid against bloatedness, flatulence, and stomach pain as dietary supplement (pepsin) or as drug for treatment of exocrine pancreatic insufficiency (pancreatic enzymes) were identified as potential α-Gal source [56] (Fig. 1).

The α-Gal syndrome has been evoked in three cases of cattle workers who presented with allergic symptoms after assisting the veterinarian during calving [57], representing the first report on α-Gal as occupational and respiratory allergen. α-Gal was detected in the amniotic fluid. Two of the workers had mainly contact urticaria limited to exposed areas of the skin, while the third one also experienced dyspnea, probably by inhalation of amniotic fluid components.

Bioprosthetic heart valves are of bovine or porcine origin. Glutaraldehyde fixation is used to ensure biocompatibility of the treated xenogeneic tissue. A study conducted by Naso and co-authors on commercially available bioprosthetic valves showed that for some products, not all α-Gal epitopes were masked by the procedure and were still detectable [58]. An association between IgE antibodies to α-Gal and a premature degeneration of bioprosthetic aortic valves is postulated in two patients who developed an allergy to α-Gal [59]. Specific IgE antibodies directed to α-Gal have also been linked recently to an increased burden of atherosclerosis and to plaques with less stable characteristics [60]. The authors hypothesize that the α-Gal epitope which is also present on mammalian glycolipids could increase the inflammatory response to dietary glycolipids in α-Gal sensitized patients. To date, the actual risk of α-Gal-sensitized patients who would require bioprosthetic heart valves or who develop an α-Gal syndrome after xenotransplantation is not known and certainly requires further monitoring.

 The abundance of natural human anti-α-Gal IgM and IgG antibodies has prompted research on their clinical application in cancer therapy [61] (Fig. 2). Glycolipids isolated from rabbit red cell membranes were injected into solid tumors. The insertion of the glycolipids into tumor cell membranes resulted in the presentation of multiple α-Gal epitopes on tumor cells. In vitro studies showed that these cells were lysed in the presence of complement and anti-α-Gal antibodies. In an Ggta1 KO mouse model, injection of glycolipids into melanoma tumors resulted in complement-mediated and antibody-dependent cell-mediated tumor regression. The local destruction of tumor cells resulted in intratumoral inflammation and a systemic anti-tumor immune response [62]. A phase 1/2a trial for solid tumor immunotherapy using a synthetic α-Gal glycolipid, AGI-134, has been initiated by BioLineRx Ltd. (Tel Aviv, Israel) (https://clinicaltrials.gov/ct2/show/NCT03593226?term=AGI-134&rank=1 ).

## Investigating Pre-Clinical Models to Better Understand Sensitization to α-Gal and Anaphylaxis to Red Meat

The absence of α-Gal is a prerequisite to allow percutaneous sensitization in humans. Mice, in contrast, express a fully functional α-1,3-galactosyltransferase and murine proteins abundantly carry α-Gal residues [63–65]. Consequently, α-Gal is a self-antigen in mice and wild-type mice develop neither IgG nor IgE directed to α-Gal [66]. In contrast, the human immune system recognizes α-Gal as non-self molecule, which consequently is capable to trigger immune reactions resulting in the production of anti-α-Gal IgG or even α-Gal specific IgE and allergy. While immune recognition of non-self proteins is well understood as is the cascade of events mounting immune responses of different qualities within the innate and adaptive immune system, characterization of immune check points and consequences in response to non-self carbohydrates is less well characterized. Thus, analyzing sensitization to α-Gal and anaphylaxis to red meat will also shed light on immune consequences following exposure to non-self carbohydrates and investigating pre-clinical models has proven to be adequate to identify underlying immune mechanisms. To this end, investigating mice deficient in α-1,3-galactosyltransferase is a suitable approach.

The abundance of IgG antibodies directed to α-Gal in human sera, described in 1983 [67], could not be associated with any disease or even symptoms. However, in 1993, Galili et al. [68], Cooper et al. [69], and Sandrin et al. [70, 71] published that anti-α-Gal antibodies in humans are associated with hyperacute graft rejection in xenotransplantation, e.g., from pigs (carrying α-Gal) to primates (deficient in α-Gal). Therefore, to understand the role of α-Gal antibodies and possibly overcome α-Gal-dependent hyperacute graft rejection, two mouse strains were generated, in 1995 by Thall et al. [64] and in 1996 by Tearle et al. [65]. Both targeted electroporated 129/sv embryonic stem cells with a construct in which the exon 9, containing almost the entire α-1,3-galactosyltransferase gene catalytic domain, had been disrupted by insertion of a neomycin resistance cassette. Effectively targeted embryonic stem cells then were injected into blastocysts to obtain either CBA × C57B16 [65] or 129SV × C57BL/6J × DBA/2J chimeric mice [64]. α-1,3-galactosyltransferase knockout mice (Ggta1 KO) were reported to be healthy despite the development of cataracts, impaired glucose tolerance, a decreased insulin sensitivity, and a more aggressive behavior [64, 65, 72, 73]. By either using an IB4 lectin staining on splenocytes [65] or anti-α-Gal antibody staining on vascular endothelial cells, both strains were shown to not express α-Gal and to not spontaneously produce anti-α-Gal antibodies [64]. Both strains were used to effectively prove that indeed high anti-α-Gal IgG and IgM antibodies mediate hyperacute graft rejection in mice within hours [74] to 8–13 days [74, 75]. In contrast, recipient Ggta1 KO mice with low anti-α-Gal antibody titers showed a prolonged graft survival of > 90 days [75]. Although Ggta1 KO mice spontaneously develop anti-α-Gal antibodies, titers remain lower than in humans [66, 75, 76]. To increase antibody titers to levels comparable to those observed in humans, a number of sensitization protocols had been developed. Many different α-Gal carrying back-bone structures were proven to be effective like KLH-Gal, *Leishmania major* promastigotes, or rabbit red blood cell membranes [66, 74, 75]. A high density of α-Gal residues and most effective sensitization was observed using rabbit red blood cell membranes intraperitoneally injected together with complete or incomplete Freund’s adjuvant (CFA or IFA respectively) [75]. The resulting α-Gal-specific antibodies (approximately 0.6% of total serum IgG) were shown to be comparable to human α-Gal-specific IgG antibodies regarding avidity (30 nM in mice, 6 nM in humans) and affinity (15 mM in mice, 50 mM in humans). Isotype distribution among antibody subclasses following α-Gal-specific sensitization in Ggta1 KO mice was also comparable to those reported for humans [75]. Investigating the density of α-Gal residues revealed striking differences between different tissues and species. Obviously, tissues most extensively analyzed are those from pigs [77] and especially rich in α-Gal are glomerulus endothelia and proximal and distal tubules in the kidney, bronchial epithelia, pancreas lobular ducts, and vascular endothelia [78]. Thus, these analyses identified pork kidneys as rich in α-Gal, already indicating their relevance for red meat allergy discovered much later [7•, 9, 10, 12, 14•, 24•, 28•, 54, 79, 80]. Interestingly but not surprisingly, α-Gal is also expressed in the thymus of α-Gal competent species [81]. Thymic expression of self antigens confers central immune tolerance by deletion of auto-reactive T cells and generation of thymic tTreg cells [82]. Accordingly, it has been postulated that all mammals synthesizing α-Gal epitopes should be tolerant to it and consequently not producing anti-Gal antibodies [83]. Therefore, the fact that Ggta1 KO mice spontaneously develop anti-α-Gal antibodies additionally confirms that they are at least not fully tolerant as orchestrated by the thymus and are capable to develop immune responses to non-self α-Gal ideally mimicking the situation in humans. Most interestingly, in Ggta1 KO mice like in humans, the initial immune response to α-Gal with induction of specific IgM and IgG antibodies is not associated to the development of any detectable dysfunction, inflammation, or disease. This is vitally important in organisms lacking intrinsic α-Gal production, since α-Gal is nearly ubiquitously expressed in the environment. Regarding α-Gal, there is strong evidence that cutaneous exposure to α-Gal in humans due to repeated tick bites is able to initiate a switch to the production of α-Gal-specific IgE. Until now, data on allergy to α-Gal in the mouse model are very limited. However, effective percutaneous sensitization to α-Gal in Ggta1 KO mice has been reported by Araujo et al [21••]. The authors either subcutaneously injected α-Gal bound to “bacteriophage Qb-virus like particles” (Qb-VLPs), each displaying 540 copies of α-Gal on its surface, once weekly for 4 weeks; injected tick saliva following the same protocol or placed one male and one female tick in parallel on the back of a mouse for 9 days using a feeding chamber. Detection of anti-α-Gal antibodies in mouse serum by ELISA using the Qb-VLPs or tick saliva as α-Gal source for coating clearly showed a relative strong induction of α-Gal-specific IgE following tick feeding and a less strong effect following subcutaneous injection of tick saliva. Interestingly, subcutaneous injection using the α-Gal-Qb-VLPs alone triggered α-Gal-specific IgG but no IgE. The authors suggests that “the salivary protein(s) bearing the α-Gal-like antigen(s) might modulate the immune response in a different way than VLP display.” Another reason might be an intrinsic Th2 immunity-promoting adjuvant function of tick saliva components. Indeed, Ohta et al. were able to prove that tick bites in mice induce skin resident IL-3 + CD4+ memory T cells predominantly being CD44 + CD62L − CD69+ [84••]. After an initial tick bite, these cells could be detected even in previously uninfested skin distant from the original feeding site and IL-3 production by these cells was proven to be necessary for the rapid recruitment of basophils to the site of a second tick bite. As basophils were repeatedly shown to be major IL-4 producers [85, 86], this mechanism might at least in part explain the Th2 triggering capacity of repeated tick bites and the consecutive production of a-Gal-specific IgE.

## Immune Responses to α-Gal in Comparison to Other Carbohydrates

Carbohydrate determinants can be found on almost all proteins and belong to the most abundant immune determinants [87]. Well-known examples are the blood group antigens or bacterial polysaccharides used as vaccination antigens, e.g., as part of the *Haemophilus influenza* type b vaccine [87]. However, until the identification of the delayed type I allergy to red meat caused by IgE directed to α-Gal, IgE recognizing carbohydrate determinants has been interpreted as mostly clinically irrelevant for type I allergy. Carbohydrate moieties as possibly relevant allergens for the first time got attention in the 1980s when Aalberse et al. reported that IgE antibodies from patients cross-react with vegetable foods, Hymenoptera venoms, and pollen, an effect which could be abolished by treatment with the strong oxidant periodate [88]. They concluded that the observed cross-reactivity is due to carbohydrates which they termed “carbohydrate cross-reactive determinants” (CCDs). Their findings could be confirmed by other groups using bromelain, a small glycopeptide isolated from the pineapple stem, which is particularly suitable because the peptide contains only two to four amino acids which are not likely to act as an antigen on their own [89]. Relevant as CCDs, since ubiquitously expressed in the environment but not as part of mammalian glycoproteins, are xylose, found in plants and parasitic worms, and core-3-linked fucose, also synthesized in insects, containing N-glycans [87]. However, interest in carbohydrate epitopes remained low, since they are usually of a low clinical significance without anaphylactic potential [90]. Next to α-Gal, only very few and much less well investigated and understood putatively clinically relevant carbohydrate allergens exist. Initially, only in Japan anaphylactic reactions to galacto-oligosaccharides (GOSs) have been reported. GOSs vary in length and type of linkage between the monomers but typically consist of a chain of 2–6 mostly galactose molecules and a terminal glucose [91]. Originally, in workers on Japanese oyster farms, a form of occupational asthma has been described for which a number of different oligosaccharitols isolated from the H-antigen of the sea squirt were identified as causative allergens [92, 93]. Interestingly, in these same oyster farm workers, later on a series of immediate-type allergic reactions after consuming a lactic acid beverage popular in Japan has been observed [94]. Jyo et al. could show that all patients had been exposed to the sea squirt working on oyster farms, that 1–3 or 1–6 linked GOS consisting of four saccharides, as identified in the anaphylaxis-causing beverage, induced positive scratch tests and histamine release assay and that the IgE antibodies directed to GOS were also cross-reactive to sea squirt antigens [94]. Most interestingly, since GOS in the meantime are often used as probiotic supplements in beverages all over the world, cases of GOS allergy triggered by other specific GOS than the aforementioned following ingestion of infant milk products or commercially available milk drinks [95, 96] have been published. Anaphylactic reactions to other foods have also been reported to depend on glycoprotein allergens; however, the data basis is mostly insufficient [97, 98••]. The knowledge about the basic mechanisms underlying immune reactions to carbohydrates is limited. Regarding immune reactions to proteins, we know that they are intracellularly cleaved and peptide fragments presented via MHCII together with costimulatory molecules then activate T cells which on their part can promote the activation of B cells. Although we do not know it for sure, at least regarding glycan moieties of glycoproteins, this mechanism most likely also applies to carbohydrate allergens. However, B1 B cells, which can be stimulated and produce antibodies without getting an activation signal from T helper cells, might also be involved. To disclose the mechanisms involved specifically in the immune reaction to α-Gal, Cretin et al. used Ggta1/T cell receptor beta chain (TCRβ) double KO mice to investigate if T cells are involved in the induction of anti-α-Gal antibodies [76]. They observed an age-dependent increase in anti-α-Gal IgG titers in Ggta1 KO mice but not in Ggta1/TCRβ double KO.

Likewise they could booster anti-α-Gal IgG titers in Ggta1 KO mice by immunization with pig cells but not in Ggta1/TCRβ double KO. In addition, treatment with anti-CD40L antibodies, which inhibits T cell-dependent B cell maturation and class switching, before immunization with pig cells could inhibit sensitization to α-Gal in Ggta1 KO mice. At least regarding α-Gal, these data indicate an important role for T cells also in immune reactions to carbohydrate allergens. However, for a fundamental and more general understanding of the mechanisms and cell types involved in immunity to carbohydrate allergens, further studies are urgently needed.

## Conclusion

Immune consequences of the exposure to non-self carbohydrates is a very important focus of research, since the cascade of immune events is much less well defined compared to immune reactions to non-self proteins. However, the description and in-depth characterization of delayed type I immediate reactions to red meat and offal demonstrates the relevance and medical consequences that the exposure to non-self carbohydrates may have. Interestingly, the exposure to non-self carbohydrates in the gastrointestinal tract apparently fails to induce adverse immune reactions while the repetitive cutaneous exposure to α-Gal through tick bites may induce α-Gal-specific IgE antibodies and a clinically relevant allergy to α-Gal. Many sources contain α-Gal such as α-Gal containing drugs, food, or volume colloids and in susceptible patients, an exposure can elicit type I allergic reactions. Importantly, so-called co- or augmentation factors may modulate the susceptibility and severity of the anaphylactic reactions. Taken together, the field warrants further attention in regard to increase our mechanistic understanding, to improve patient risk assessment, patient care, but also to possibly introduce prevention of disease development. To this end, a deeper look into (i) tick biology and (ii) the transmission of adjuvants and (iii) α-Gal containing cells, proteins, and lipids; (iv) the translation of tick-derived information into immune consequences within the skin; (v) the establishment of IgE production; and (vi) the consecutive regulation of anaphylactic responses following exposure to α-Gal are categories of investigations necessary and each asks for detailed analyses and single-step characterization. Based on these analyses, new concepts will arise that not only allow us to better understand the “immune digestion” of α-Gal containing proteins and lipids but also to extrapolate the basic findings to carbohydrate immunology per se in regard to its recognition, carbohydrate-induced immune responses, the regulation of immune tolerance towards carbohydrates, and the longevity of carbohydrate-directed immune responses.
